# Evaluation of regulatory genetic variants in *POU5F1* and risk of congenital heart disease in Han Chinese

**DOI:** 10.1038/srep15860

**Published:** 2015-10-28

**Authors:** Yuan Lin, Chenyue Ding, Kai Zhang, Bixian Ni, Min Da, Liang Hu, Yuanli Hu, Jing Xu, Xiaowei Wang, Yijiang Chen, Xuming Mo, Yugui Cui, Hongbing Shen, Jiahao Sha, Jiayin Liu, Zhibin Hu

**Affiliations:** 1 State Key Laboratory of Reproductive Medicine, Nanjing Medical University, Nanjing 210029, China; 2 Department of Epidemiology and Biostatistics and Key Laboratory of Modern Toxicology of Ministry of Education, School of Public Health, Nanjing Medical University, Nanjing 211166, China; 3 Clinical Center of Reproductive Medicine, the First Affiliated Hospital of Nanjing Medical University, Nanjing 210029, China; 4 Department of Cardiothoracic Surgery, Nanjing Children’s Hospital, Nanjing Medical University, Nanjing 210008, China; 5 Department of Thoracic and Cardiovascular Surgery, The First Affiliated Hospital of Nanjing Medical University, Nanjing 210029, China; 6 Department of Histology and Embryology, Nanjing Medical University, Nanjing 210029, China

## Abstract

OCT4 is a transcription factor of the POU family, which plays a key role in embryonic development and stem cell pluripotency. Previous studies have shown that Oct4 is required for cardiomyocyte differentiation in mice and its depletion could result in cardiac morphogenesis in embryo. However, whether the genetic variations in OCT4 coding gene, *POU5F1*, confer the predisposition to congenital heart disease (CHD) is unclear. This study sought to investigate the associations between low-frequency (defined here as having minor allele frequency (MAF) between 0.1%–5%) and rare (MAF below 0.1%) variants with potential function in *POU5F1* and risk of CHD. We conducted association analysis in a two-stage case-control study with a total of 2,720 CHD cases and 3,331 controls in Chinese. The low-frequency variant rs3130933 was observed to be associated with a significantly increased risk of CHD [additive model: adjusted odds ratio (OR) = 2.15, adjusted *P* = 3.37 × 10^−6^]. Furthermore, luciferase activity assay showed that the variant A allele led to significantly lower expression levels as compared to the G allele. These findings indicate for the first time that low-frequency functional variant in *POU5F1* may contribute to the risk of congenital heart malformations.

Congenital heart disease (CHD) refers to the structural, functional or metabolic abnormalities of heart or major blood vessels that arise during embryogenesis. It is currently the most common cause of birth defect worldwide, accounted for 28% of all major congenital anomalies^1^. Epidemiologically, the prevalence of CHD is estimated to be 8 per 1,000 live births, and the incidence is higher if fetuses that do not survive to term are included^1^,^2^,^3^. CHD can be classified into three broad categories: cyanotic heart disease, left-sided obstruction defects and septation defects. Septation defects are most common which include atrial septal defect (ASD), septation of the ventricle (VSD) as well as other subtypes^4^.

The origin of CHD is quite complex and it may result from a multifactorial inheritance model^5^. Over the past decade, some underlying causative factors of CHD have been identified, including genetic abnormalities^4^,^6^, unfavorable environmental exposure, as well as maternal lifestyle^7^,^8^. Genetically, CHD is a very heterogeneous disease that involves a multitude of susceptibility genes with low-penetrance factors (common variants) or intermediate-penetrance factors (low-frequency and rare variants). There has been an identification of more than 50 human genes involved in isolated CHD^9^. However, the etiology of CHD is still incompletely understood so far. Cardiogenesis is initiated with the formation of mesodermal multipotent cardiac progenitor cells and is governed by cross-talk between developmental cues emanating from endodermal, mesodermal and ectodermal cells. The molecular and transcriptional machineries that direct the specification and differentiation of these cardiac precursors are part of an evolutionarily conserved programme that includes the Nkx-, Gata-, Hand-, T-box- and Mef2 family of transcription factors^10^. A cascade of transcription factors coding genes crucial for cardiac progenitor lineages (eg, *NKX2–5*, *GATA4*, *TBX5*, *HAND1*, and *MEF2C*) are well known to be involved in the development of specific defects in heart development, such as cardiac septation or outflow tract malformations^4^. These genes were discovered to be with high-penetrance mutations that caused syndromic or isolated CHDs^11^,^12^,^13^.

*POU5F1* (also known as *OCT4*) is a homeodomain-containing transcription factor coding gene in the POU family. With a critical function in embryonic development and stem cell pluripotency, *POU5F1* is highly expressed in embryonic stem cells^14^. A series of *in vitro* studies have inferred many roles of Oct4 between the prestreak and headfold stage, including regulating neural versus mesendoderm differentiation as well as promoting cardiomyocyte and neuronal differentiation^15^. Oct4 expression in the blastocyst is required for heart development and the depletion of Oct4 is found to result in random heart tube orientation and thin ventricular walls at different stages of mice embryonic development^15^,^16^.

However, to date, no study on the possible correlation between variations of *POU5F1* and CHD risk has been published. Using our existing CHD genome-wide association study (GWAS) data^17^, we found null associations between common [minor allele frequency (MAF) ≥5%] single nucleotide polymorphisms (SNPs) in *POU5F1* and CHD risk (data not shown). Therefore, in this study, we investigated the potentially functional low-frequency (MAF between 0.1%–5%) and rare variants (MAF less than 0.1%) in coding or regulatory regions of *POU5F1* to find out whether these variants influence susceptibility to CHD. After excluding SNPs with MAF ≥0.05 in Chinese Han population, 9 variants located in regulatory regions remained. We then used a web-based analysis tool, RegulomeDB (http://regulome.stanford.edu/index), to predict the function of these SNPs. The variants rs3130933 and rs17190811 with the score of 1f and 2b are more likely located within regulatory elements. Therefore, we finally included these 2 SNPs in our study (Methods). To reduce the heterogeneity between different phenotypes, we only included the most common subtypes, ASD, VSD and ASD combined VSD, in the GWAS scan as well as in the current study. Different phenotypes of CHD originated from different part of heart, and some recent studies identified that genetic etiology of CHD could have a considerable degree of phenotypic specificity^18^,^19^. Therefore, we also performed stratification analysis in different diagnostic groups.

## Results

The selected characteristics of the CHD cases and non-CHD controls in two stages were described in Supplementary Table S1. We showed the genotype distributions of these two variants (rs3130933 and rs17190811) in cases and controls in Table 1. These two variants followed Hardy-Weinberg equilibrium in the controls in both two stages (stage I: *P* = 1.00 for rs3130933, *P* = 0.06 for rs17190811; stage II: *P* = 1.00 for rs3130933, *P* = 0.28 for rs17190811). In the first-stage association study, both of these two variants exhibited significant associations with the risk of CHD with adjustment for sex (rs3130933: adjusted OR = 2.19, 95% CI = 1.34–3.58, adjusted *P* = 1.67 × 10^−3^ in additive model; rs17190811: adjusted OR = 1.58, 95% CI = 1.28–1.96, adjusted *P* = 2.82 × 10^−5^ in additive model) after performing multiple testing corrections with the Bonferroni single-step method (corrected α: 0.05/2 = 0.025) (Table 1). To further validate such associations, we performed another association study and the results showed that rs3130933 mutant allele was consistently associated with the increased risk of CHD (adjusted OR = 2.13, 95% CI = 1.39–3.28, adjusted *P* = 5.68 × 10^−4^ in additive model). However, no significant association was observed for the variant rs17190811 in stage II (adjusted OR = 1.08, 95% CI = 0.87–1.33, adjusted *P* = 5.07 × 10^−1^ in additive model) (Table 1). After Bonferroni correction (corrected α: 0.05/2 = 0.025), rs3130933 still remained significant association with CHD risk.

The combined analysis with samples from two stages showed a significantly increased CHD risk for the variant rs3130933 (AG *vs.* GG: adjusted OR = 2.09, 95% CI = 1.51–2.90, adjusted *P* = 1.10 × 10^−5^; AG/AA *vs.* GG: adjusted OR = 2.14, 95% CI = 1.54–2.97, adjusted *P* = 5.27 × 10^−6^; additive model: adjusted OR = 2.15, 95% CI = 1.56–2.97; adjusted *P* = 3.37 × 10^−6^) (Table 1). Furthermore, we evaluated the associations of rs3130933 in the different diagnostic groups of ASD, VSD and ASD combined VSD using combined case-control sample sets and the results of different subtypes were shown in Table 2. We also evaluated the heterogeneity of association effects between different subtypes using the Cochran’s Q statistic test and no significant heterogeneity was observed among the effect sizes of different subtypes (*P* = 0.519 for heterogeneity test) (Table 2).

**Figure 1:**
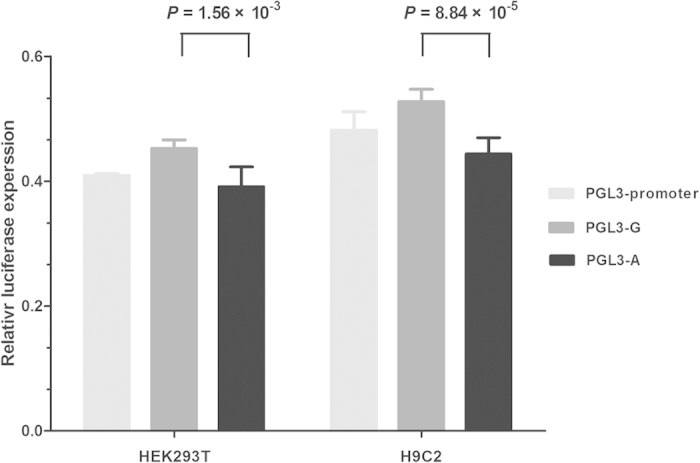
Luciferase reporter assays of rs3130933 G/A in HEK293T and H9C2 cell lines. Each transfection was performed with pRL-SV40 plasmids as normalized controls. Luciferase expression is significantly decreased in the minor A allelic construct compared with the major G construct in HEK293T and H9C2 cells. The actual values in HEK293T cells are as follows: pGL3-promoter, 0.409 ± 0.003; pGL3-G, 0.452 ± 0.013; and pGL3-A, 0.391 ± 0.029. The values in H9C2 cells were the following: pGL3-promoter, 0.482 ± 0.027; pGL3-G, 0.528 ± 0.018; and pGL3-A, 0.444 ± 0.024. Each value represents the mean ± SD of 2 experiments, with each experiment performed in triplicate.

Using RegulomeDB database, we predicted that rs3130933 is in regulatory elements and likely to affect binding of transcriptional factors (Supplementary Table S2). To understand the regulatory role of rs3130933 and interpret its direction of effect as found in the association with CHD, we assayed luciferase activity after targeted variation at rs3130933 in HEK293T and H9C2 cell lines. We expected that variant allele (A) would reduce expression levels as compared to the reference allele (G). We generated two luciferase reporter plasmids containing rs3130933 G and A allele, respectively, and used pRL-SV40 plasmids as an internal control to normalize the transfections. As expected, a plasmid containing the mutant A allele displayed significantly lower luciferase expression than the wild type G allele in HEK293T cells and H9C2 cells (0.391 *vs.* 0.452, *P* = 1.56 × 10^−3^; 0.444 vs. 0.528, *P* = 8.84 × 10^−5^, respectively) (Fig. 1).

## Discussion

In this two-stage case-control study with a total of 2,720 CHD cases and 3,331 controls from Han Chinese population, we investigated the associations of two potentially functional variants in *POU5F1* with risk of CHD. We identified that the variant allele A of rs3130933 increased the risk of CHD significantly. We estimated the statistical power for rs3130933 according to the sample size and association effect, and our study could reach 92.1% of the power. After checking our CHD GWAS data, we found that the low-frequency variant rs3130933 did not show up in original data as well as imputation data. We further conducted bioinformatics analysis using RegulomeDB database which showed that the variant rs3130933 is very likely located in the target site of transcriptional factors. We hypothesized that rs3130933 mutant allele (A) might reduce *POU5F1* expression at the transcriptional level and our results of luciferase reporter gene assay verified this hypothesis. These results accentuate the potentially important involvement of functional low-frequency variants of *POU5F1* in the origin of CHD.

CHD is a complex process which is caused by developmental corruption in early embryogenesis leading to abnormalities in heart’s structure or function. OCT4, as a key transcription factor, has been established as a hub of the signaling network that maintains pluripotency and normal embryonic development^20^,^21^,^22^. In the stratified analysis, we did not observe heterogeneity of association strengths for rs3130933 among 3 different subtypes, which suggested that variation of *POU5F1* could lead to defects of various parts of heart in the process of development. Some studies have showed that Oct4 triggers expression of cardiac specific genes through Smad2/4 and TGFβ at early stages of differentiation^16^. Oct4 deficient in blastocysts leads to defect in cardiac morphogenesis in embryo, featuring trabeculation reduction and myofibrillogenesis impairment of ventricle^16^. It has also been reported that Oct4 is associated with a varied set of proteins, the majority of which show an early lethal phenotype when mutated. A fraction of the human orthologs of Oct4-associated proteins is associated with inherited developmental disorders^23^.

The significant low-frequency variant rs3130933 identified in our study is located in the 3′ near gene of *POU5F1*. The results of our reporter gene assay indicated that rs3130933 mutant A allele resulted in significantly low transcription activity and *POU5F1* expression level. According to the RegulomeDB database, this variant locates in regulatory elements and has potential regulation function (Supplementary Table S2). ChIP-seq data suggests that rs3130933 is at the binding site of nuclear transcription factor Y subunit beta (NFYB) and CCAAT/enhancer binding protein beta (CEBPB) in GM12878 (B-lymphocyte, lymphoblastoid) cell line (Supplementary Fig. S1). The eukaryotic transcription factor NF-Y is a heterotrimeric complex, which specifically recognizes the regulatory CCAAT element found in either orientation in the proximal and distal enhancer regions of many genes^24^,^25^. CEBP family of transcription factors contain basic leucine zipper (bZIP) domain, and they have been proved to be activated in the epicardium in both heart development and the injury response^26^. ChIP-seq data also shows that this variant is likely located at site of H3k4me1 histone modification mark in fetal heart tissue (Supplementary Fig. S1). H3K4me1 histone marks are often found near enhancers and downstream of transcription starts. As the expression experiment had shown, the variant A allele could reduce transcription activity significantly, which may due to its effect on transcriptional factors binding. OCT4 acts as a hub of the signaling network. The change of OCT4 dosage would influence expression of cardiac specific genes^16^, which may be a possible underlying mechanism for the observed association between rs3130933 and the risk of CHD. Additionally, according to RegulomeDB database, rs3130933 was identified to be the transcript expression quantitative trait loci (transcript-QTL) and exon-QTL of HLA-C coding gene in lymphoblastoid cell lines as a cis-acting element. However, these results are very preliminary and merit further investigations.

In conclusion, the present study evaluated low-frequency variants with potential function in *POU5F1* and confirmed that rs3130933 was associated with CHD in Han Chinese populations. Report gene assay further provided evidence that this variant may increase the risk of CHD through affecting transcription activity. Although luciferase experiment showed a small effect of the variant on the expression, they suggested potential important regions in genome. In addition, the variant rs3130933 is located in the MHC region on chromosome 6, which increases the complexity of results explanation because of strong linkage disequilibrium in this region. Therefore, further functional studies including analysis on cell lines or tissues may help to validate our findings and association or sequencing study of this region should be facilitated in other populations, particularly non-Asian populations. Additionally, the current study was designed based on candidate gene method which was difficult to analyze underlying mechanism of CHD completely, particularly for the new genes involved in CHD. Further research like gene based analysis is needed to clarify the genetic risk factors of CHD systematically.

## Methods

### Ethics Statement

The study conformed to the principles outlined in the Declaration of Helsinki and was approved by the institutional review board of Nanjing Medical University (FWA00001501). The design and performance of current study involving human subjects were clearly described in a research protocol. All participants over the age of 18 would complete the informed consent in writing before taking part in this research. For the participants under the age of 18, parental written consent was obtained.

### Study Population

The GWAS scan included 945 sporadic ASD, VSD and ASD/VSD cases and 1,246 controls recruited from the First Affiliated Hospital of Nanjing Medical University and the Affiliated Nanjing Children’s Hospital of Nanjing Medical University (Nanjing, China) between March 2006 and March 2009 14.

A two-stage case-control study was designed to evaluate the associations between potentially functional low-frequency and rare variants in *POU5F1* and the risk of CHD. A total of 2,720 CHD cases and 3,331 non-CHD controls were enrolled between March 2009 and March 2014. For stage I, 1,309 cases and 1,491 controls were recruited from the First Affiliated Hospital of Nanjing Medical University, Nanjing, China. Stage II consisted of 1,411 cases and 1,840 controls recruited from the Affiliated Nanjing Children’s Hospital of Nanjing Medical University, Nanjing, China. All subjects were genetically unrelated ethnic Han Chinese. CHD were diagnosed based on echocardiography, some of which were further confirmed by cardiac catheterization and/or surgery. CHD patients who manifested clinical features of developmental syndromes, multiple major developmental anomalies or known chromosomal abnormalities were excluded. Exclusion criteria also included maternal diabetes mellitus, phenylketonuria, maternal teratogen exposures (e.g., pesticides and organic solvents), and maternal therapeutic drugs exposures during the intrauterine period. All controls were non-CHD outpatients from the same geographical area over the same time period. Controls with congenital anomalies or cardiac disease were excluded. For each participant, approximately 2-ml whole blood was obtained to extract genomic DNA for genotyping analysis.

### SNP Selection

We searched potentially functional SNPs in coding regions (nonsynonymous and nonsense variants) and regulatory regions (promoter, 5′-UTR, 3′-UTR, 3′ near gene and 5′ near gene) in *POU5F1* gene in the dbSNP database (http://www.ncbi.nlm.nih.gov/projects/SNP/). Those SNPs with MAF ≥ 0.05 in Chinese Han population (CHB) were excluded and 9 low-frequency (0.001 ≤ MAF < 0.05) or rare variants (MAF < 0.001) remained. We found that all of the 9 variants are located in regulatory regions. So we used a web-based analysis tool, RegulomeDB, to predict the function of these SNPs (http://regulome.stanford.edu/). The variants with the score of 1 or 2 were suggested to be in regulatory elements and more likely to affect binding of transcriptional factors. The variants rs3130933 and rs17190811 with the score of 1f and 2b were finally included in the study and their primary information was described in Supplementary Table S2.

### Genotyping

Genotyping analyses were performed using the TaqMan allelic discrimination assay on the platform of 7900HT Real-time PCR System (Applied Biosystems). The information of the primers and probes were provided in Supplementary Table S3. A series of methods were used to control the quality of genotyping: (i) case and control samples were mixed on each plate; (ii) the laboratory technicians who performed the genotyping were blinded to the case/control status of the samples; (iii) two water controls were used in each 384-well format as blank control; (iv) 5% of the samples were randomly selected for repeat genotyping. The genotyping results were determined by using SDS 2.3 Allelic Discrimination Software (Applied Biosystems). Genotyping error rates of these two SNPs by Taqman were shown in Supplementary Table S4.

### Luciferase plasmids construct and site-directed mutagenesis

To construct the luciferase reporter plasmids containing the variant rs3130933, we amplified the 822-bp fragment of *POU5F1*, which contains the A allele of rs3130933, by PCR from genomic DNA (Supplementary Fig. S2A). The primers were CCCTCGAGCAGAGCCAGGAATAAAA (sense) and TGACGCGTCTCACTTCACTGCACTG (antisense). Purified PCR products and pGL3-promoter vector (Promega) were both cleaved by Mlu I and Xho I enzymes (New England BioLabs), and then were ligated by T4 DNA ligase (New England BioLabs) to the recombinant constructs (Supplementary Fig. S2B). The construction of the recombinant plasmid was shown in Supplementary Fig. S2C. Four recombinant plasmids were sequenced and the sequencing results were 100% correct. The plasmid containing A allele was transformed to E. coli DH5α™ competent cells (Vazyme Biotech) and cultured for 12 hours. Then we used Mutagenesis Kit to generate the corresponding G allele plasmids. The new plasmids were transformed to E. coli DH5α™ competent cells and cultured for 12 hours. Different monoclonal were selected for liquid bacterial cultivation. After sequencing, we found the rate of successful mutagenesis of G allele was 50%. The sequencing results of wild type and mutant recombinant plasmids were shown in Supplementary Fig. S3.

### Transient transfection and luciferase assays

Human embryonic kidney 293T (HEK293T) cells and rat cardiac myocyte (H9C2) cells were maintained in DMEM medium with 10% heat-inactivated fetal bovine serum (Gibco) and 50 ug/ml streptomycin (Gibco) at a 37 °C incubator supplemented with 5% CO_2_. HEK293T cells or H9C2 cells (1 × 10^5^) were seeded in 24-well culture plates and incubated for 24 h before transfection. Transfections were performed using Lipofectamine 2000 according to the manufacturers’ protocol (Invitrogen). The pRLSV40 containing *Renilla reniformis* luciferase was cotransfected to standardize transfection efficiency as a normalizing control. After another 24 hours of culture, the transfected cells were collected and assayed for luciferase activity with the Dual-Luciferase Reporter Assay System (Promega). Two independent transfection experiments were performed, and each luciferase assay was carried out in triplicate. More details were described in Supplementary Methods.

### Statistical analysis

Hardy-Weinberg equilibrium for the distribution of each variant was evaluated using the goodness of-fit χ^2^ test by comparing the observed genotype frequencies with the expected ones in the controls.

Statistical power was evaluated according to the sample size and association effect in additive model by using PS software (Power and the Sample Size Calculations version 3.1.2).

To evaluate the associations between the genotypes and CHD risk, odds ratios (ORs) and 95% confidence intervals (CIs) were calculated by unconditional logistic regression analysis with adjustment for sex.

The heterogeneity between subgroups was assessed with the Chi-square-based Cochran’s Q test which is computed by summing the squared deviations of each study’s effect estimate from the overall effect estimate, weighting the contribution of each study by its inverse variance. If *P* value > 0.05, we could not reject the homogeneity hypothesis which assumed that the estimated effect sizes only differ by sampling error. In contrast, if *P* value ≤ 0.05, homogeneity assumption was rejected which meant that between-studies variability existed. All statistical tests were 2 tailed, with *P* ≤ 0.05 set as the significance level. All analyses were performed using PLINK 1.07 (http://pngu.mgh.harvard.edu/~purcell/plink/).

## Additional Information

**How to cite this article**: Lin, Y. *et al.* Evaluation of regulatory genetic variants in *POU5F1* and risk of congenital heart disease in Han Chinese. *Sci. Rep.* **5**, 15860; doi: 10.1038/srep15860 (2015).

## Supplementary Material

Supplementary Materials

## Figures and Tables

**Table 1:** Association results for the 2 *POU5F1* variants in stage I, stage II and combined sample set.

	SNP	Genotype	Cases n (%)	Controls n (%)	MAF^a^		Crude OR (95%CI)	Adjusted OR^b^ (95%CI)	Adjusted *P*^b^
					Cases	Controls			
**Stage I (1309 cases *vs.* 1491 controls)**	rs3130933	GG	1260 (96.48)	1461 (98.32)	0.018	0.008	1.00	1.00	
		AG	45 (3.45)	25 (1.68)			**2.09 (1.27–3.42)**	**2.13 (1.29–3.50)**	**3.02 × 10**^**−3**^
		AA	1 (0.07)	0 (0.00)			–	–	–
		AG+AA	46 (3.52)	25 (1.68)			**2.13 (1.30–3.49)**	**2.18 (1.33–3.59)**	**2.08 × 10**^**−3**^
		Additive					**2.14 (1.32–3.48)**	**2.19 (1.34–3.58)**	**1.67 × 10**^**−3**^
	rs17190811	AA	1082 (82.91)	1309 (88.44)	0.087	0.058	1.00	1.00	
		AT	220 (16.86)	170 (11.49)			**1.57 (1.26–1.94)**	**1.58 (1.27–1.96)**	**4.06 × 10**^**−5**^
		TT	3 (0.23)	1 (0.07)			3.63 (0.38–34.94)	2.58 (0.27–24.83)	4.13 × 10^−1^
		AT+TT	223 (17.09)	171 (11.56)			**1.58 (1.27–1.96)**	**1.59 (1.28–1.97)**	**3.11 × 10**^**−5**^
		Additive					**1.58 (1.28–1.95)**	**1.58 (1.28–1.96)**	**2.82 × 10**^**−5**^
**Stage II (1411 cases *vs.* 1840 controls)**	rs3130933	GG	1345 (96.00)	1805 (98.15)	0.020	0.009	1.00	1.00	
		AG	55 (3.93)	34 (1.85)			**2.17 (1.41–3.35)**	**2.07 (1.34–3.21)**	**1.07 × 10**^**−3**^
		AA	1 (0.07)	0 (0.00)			–	–	–
		AG+AA	56 (4.00)	34 (1.85)			**2.21 (1.44–3.40)**	**2.12 (1.37–3.28)**	**7.25 × 10**^**−4**^
		Additive					**2.22 (1.45–3.40)**	**2.13 (1.39–3.28)**	**5.68 × 10**^**−4**^
	rs17190811	AA	1230 (87.86)	1617 (88.60)	0.061	0.058	1.00	1.00	
		AT	168 (12.00)	205 (11.23)			1.08 (0.87–1.34)	1.08 (0.87–1.35)	4.76 × 10^−1^
		TT	2 (0.14)	3 (0.17)			0.88 (0.15–5.25)	0.88 (0.15–5.37)	8.92 × 10^−1^
		AT+TT	170 (12.14)	208 (11.40)			1.07 (0.87–1.33)	1.08 (0.87–1.34)	4.88 × 10^−1^
		Additive					1.07 (0.87–1.32)	1.08 (0.87–1.33)	5.07 × 10^−1^
**Combined sample set (2720 cases *vs.* 3331 controls)**	rs3130933	GG	2605 (96.23)	3266 (98.23)	0.019	0.009	1.00	1.00	
		AG	100 (3.70)	59 (1.77)			**2.13 (1.53–2.94)**	**2.09 (1.51–2.90)**	**1.10 × 10**^**−5**^
		AA	2 (0.07)	0 (0.00)			–	–	–
		AG+AA	102 (3.77)	59 (1.77)			**2.17 (1.57–3.00)**	**2.14 (1.54–2.97)**	**5.27 × 10**^**−6**^
		Additive					**2.17 (1.58–3.00)**	**2.15 (1.56–2.97)**	**3.37 × 10**^**−6**^

^a^Minor allele frequency (MAF).

^b^Adjusted for sex. Significant values (*P* < 0.05) are in bold.

**Table 2:** Summary of stratified analyses by gender and subtypes of CHD for the variant rs3130933.

Diagnostic groups	Genotype	Cases n (%)	Controls n (%)	MAF^a^		Crude OR (95%CI)	Adjusted OR^b^ (95%CI)	Adjusted *P*^b^	Heterogeneity*P*^c^
				Cases	Controls				
ASD	GG	726	3266	0.015	0.009	1.00	1.00		
	AG	21	59			1.60 (0.97–2.65)	1.51 (0.90–2.52)	1.19 × 10^−1^	
	AA	1	0			–	–	–	
	AG+AA	22	59			**1.68 (1.02–2.76)**	1.59 (0.96–2.64)	7.25 × 10^−2^	
	Additive					**1.73 (1.07–2.80)**	**1.66 (1.01–2.71)**	**4.38 × 10**^**−2**^	
VSD	GG	1458	3266	0.018	0.009	1.00	1.00		
	AG	63	59			**2.39 (1.67–3.43)**	**2.32 (1.62–3.33)**	**5.21 × 10**^**−6**^	0.519
	AA	1	0			–	–	–	
	AG+AA	64	59			**2.43 (1.70–3.48)**	**2.36 (1.65–3.39)**	**3.03 × 10**^**−6**^	
	Additive					**2.44 (1.71–3.48)**	**2.37 (1.66–3.39)**	**2.16 × 10**^**−6**^	
ASD/VSD	GG	421	3266	0.021	0.009	1.00	1.00		
	AG	16	59			**2.10 (1.20–3.69)**	**2.09 (1.19–3.68)**	**1.04 × 10**^**−2**^	
	AA	0	0			–	–	–	
	AG+AA	16	59			**2.10 (1.20–3.69)**	**2.09 (1.19–3.68)**	**1.04 × 10**^**−2**^	
	Additive					**2.10 (1.20–3.69)**	**2.09 (1.19–3.68)**	**1.04 × 10**^**−2**^	

^a^Minor allele frequency (MAF).

^b^Adjusted for sex. Significant values (*P* < 0.05) are in bold.

^c^*P* values for heterogeneity test between groups using ORs estimated in adjusted additive model.
