# Supplementary material for: Evaluation of regulatory genetic variants in POU5F1 and risk of congenital heart disease in Han Chinese
Source: Sci Rep. 2015 Oct 28;5:15860. doi: 10.1038/srep15860 (PMC4623744; doi:10.1038/srep15860)
Supplement: Supplementary Materials [file srep15860-s1.pdf]

Supplementary Information

Evaluation of regulatory genetic variants in *POU5F1* and risk of congenital heart disease in Han Chinese

Yuan Lin[1,2]*, Chenyue Ding[1,3]*, Kai Zhang[1,2], Bixian Ni[1,2], Min Da[4] , Liang Hu[4] , Yuanli Hu[4] ,Jing Xu[5], Xiaowei Wang[5], Yijiang Chen[5], Xuming Mo[4], Yugui Cui[3], Hongbing Shen[1,2], Jiahao Sha[1,6], Jiayin Liu[1,3], Zhibin Hu[1,2]

[1]State Key Laboratory of Reproductive Medicine, Nanjing Medical University, Nanjing 210029, China,

[2]Department of Epidemiology and Biostatistics and Key Laboratory of Modern Toxicology of Ministry of Education, School of Public Health, Nanjing Medical University, Nanjing 211166, China,

[3]Clinical Center of Reproductive Medicine, the First Affiliated Hospital of Nanjing Medical University, Nanjing 210029, China,

[4]Department of Cardiothoracic Surgery, Nanjing Children's Hospital, Nanjing Medical University, Nanjing 210008, China,

[5]Department of Thoracic and Cardiovascular Surgery, The First Affiliated Hospital of Nanjing Medical University, Nanjing 210029, China,

[6]Department of Histology and Embryology, Nanjing Medical University, Nanjing 210029, China.

Correspondence and requests for materials should be addressed to Z.H. (zhibin_hu@njmu.edu.cn) or J.L. (jyliu_nj@126.com)

* These authors contributed equally to this work.

Supplementary Methods

Efficiency of transient transfection

In the preliminary experiments, we found that it is appropriate to transfect 500 ng of PGL3-G, PGL3-A and empty PGL3 vector separately with 5 ng pRLSV40 using 10μL transfection reagent lipofectamine 2000 per well. The luciferase activity of Firefly and Renilla exceeded 4×10^{-5} and 8×10^{-5} per well respectively.

In-vitro cell culture

The 293T and H9C2 cells were seeded in 24-well plates (100,000 cells per well) containing DMEM medium with 10% heat-inactivated fetal bovine serum and 50 ug/ml streptomycin 24h before transfection. Because these two cells are adherent cells, we didn't count the cells before and after transfection. These cells covered about 80% of each well after 24 hours of incubation before transfection and covered about 90% of each well after 24 hours of transfection.

Supplementary Figure S1. Genome Browser image of rs130933 region on human assembly hg19

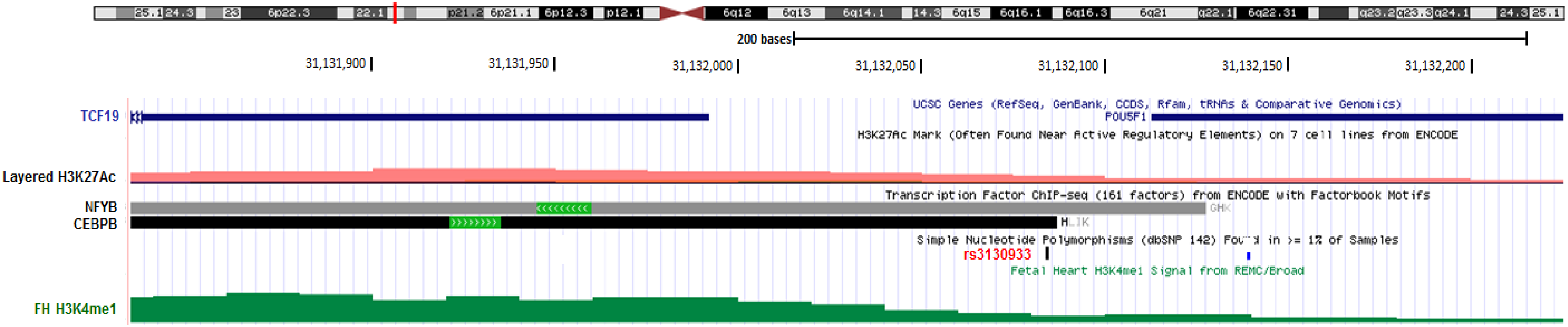

Supplementary Figure S2. Gel of amplicon of the 822-bp fragment of *POU5F1* A) PCR products of the 822-bp fragment of *POU5F1* containing rs3130933 A allele from genomic DNA. The same DNA sample was divided into four to amplify using PCR. B) PCR products of the 822-bp fragment of *POU5F1* containing rs3130933 A allele from PGL3-A recombinant constructs. The four recombinant plasmids were generated by four PCR products respectively. C) Construction of the recombinant plasmid. Rs3130933 mutant allele was shown in red font.

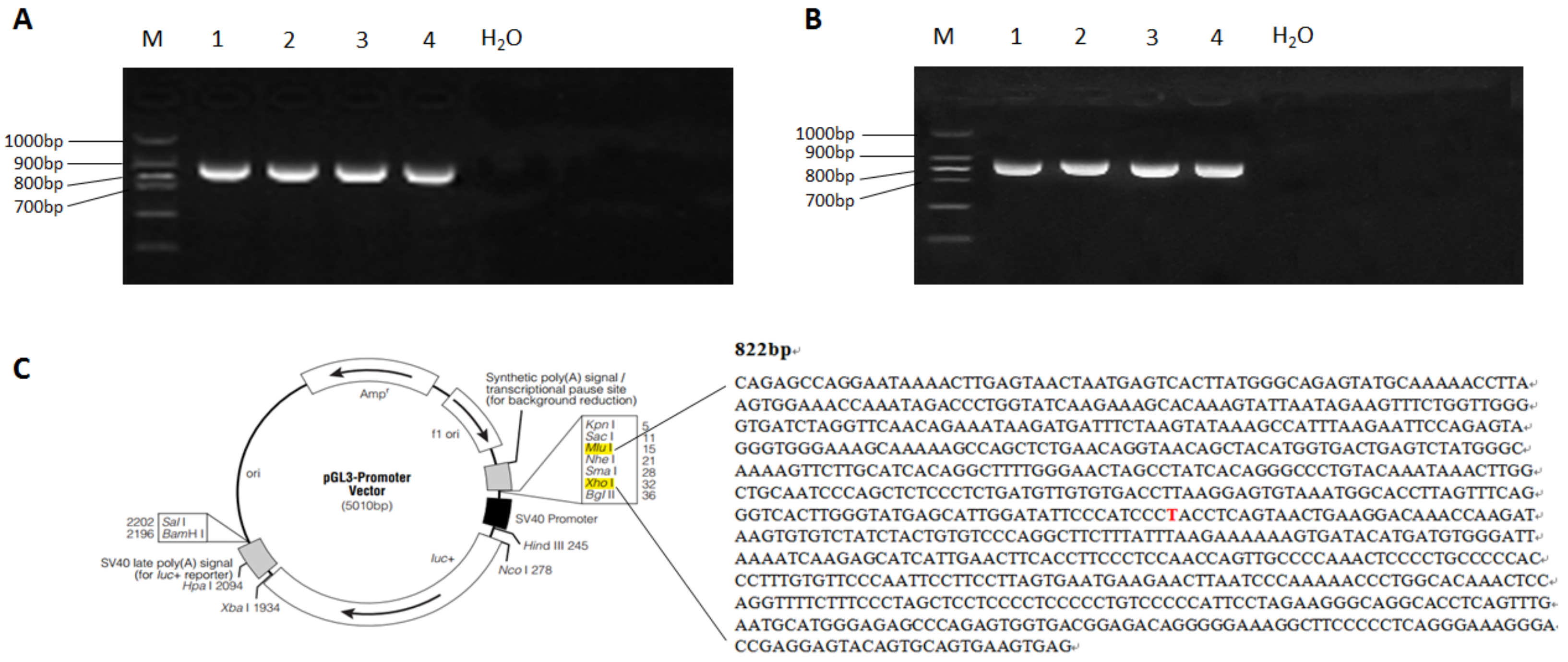

Supplementary Figure S3. The sequencing results of PGL3-G and PGL3-A recombinant constructs A) The sequencing results of the first 600bp of the 822-bp fragment of *POU5F1* containing rs3130933. B) The sequencing results of the last 600bp of the 822-bp fragment of *POU5F1* containing rs3130933.

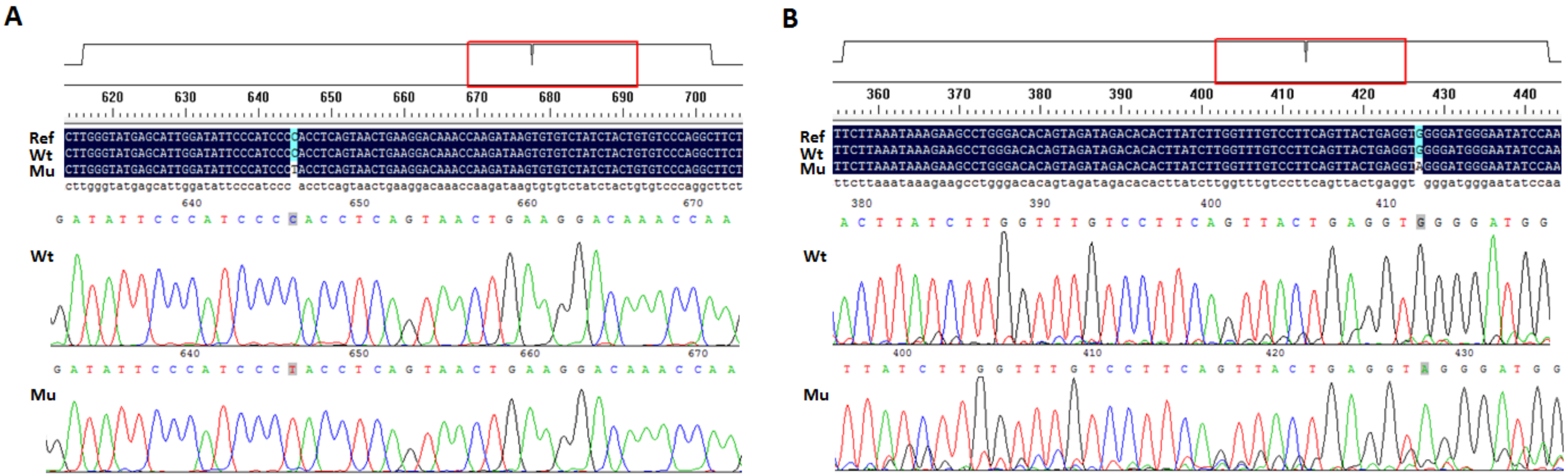

Supplementary Table S1: Characteristics of CHD cases and controls in stage I and stage II

	Stage I		Stage II	
**Variables**	**Case (N=1309)**	**Control (N=1491)**	**Case (N=1411)**	**Control (N=1840)**
Age (mean ±SD)	7.01 ±13.84	4.48 ±5.78	2.33 ±2.51	2.38 ±2.52
Sex				
Male	604	883	695	1149
Female	705	608	716	691
Phenotype				
ASD	535		217	
VSD	681		847	
ASD/VSD	93		347	
Call rate (%)				
rs3130933	99.77%	99.66%	99.29%	99.95%
rs17190811	99.69%	99.26%	99.22%	99.18%

ASD: ostium secundum atrial septal defec.

VSD: ventricular septal defect.

Supplementary Table S2. Primary information for 2 functional variants in *OCT4* gene

Chr. (cytoband)	Gene	SNP	Alleles [a]	Position (bp) [b]	Location	Regulome DB Score [c]	Predicted function [d]
6p21.33	*POU5F1*	rs3130933	G/A	31132085	3' near gene	1f	protein binding
		rs17190811	A/T	31131837	3' near gene	2b	protein binding

[a] Major/minor allele.

[b] Derived from an online tool-SNPinfo (http://snpinfo.niehs.nih.gov/snpfunc.htm).

[c] Derived from an online tool- Regulome DB (http://regulome.stanford.edu/).

[d] The scoring system of RegulomeDB was developed based on functional confidence of a variant. Lower scores indicate increasing evidence for a variant to be located in a functional region. Variants that are known eQTLs for genes, and thus have been shown to be associated with expression, as most likely to be significant are labeled as Category 1. Sub-categories within Category 1 indicate additional annotations from the most confident (1a, which has TF binding, a motif for that TF, and a DNase footprint) to the least confident (1f, which has only TF binding or a DNase peak).

Supplementary Table S3. Information of primers and probes

rs3130933		rs17190811	
F-primer	TGTGGACTCTAGAATGGACTTCCA	F-primer	GGGACACAGTAGATAGACACACTTATCTTG
R-primer	TGTCAGATTAGGACCATCTCCATCT	R-primer	CTTAGTTTCAGGGTCACTTGGGTATG
FAM-probe	FAM-AGTGACTTTACTGTCCTC-MGB	FAM-probe	FAM-TACTGAGGTGGGGATG-MGB
HEX-probe	HEX-AAGTGACTTTAATGTCC-MGB	HEX-probe	HEX-TACTGAGGTAGGGATGG-MGB

Supplementary Table S4. Genotyping error rates of these two SNPs by Taqman

SNP	Repeat genotyping samples	Inconsistent samples (%)	Consistent rate
rs3130933	303	0 (0.00%)	100%
rs17190811	303	1 (0.33%)	99.67%
